# Insights into Homogeneous Bulk Boron Doping at the Tetrahedral Site of NCM811 Cathode Materials: Structure Stabilization by Inductive Effect on *TM*‐O‐B Bonds

**DOI:** 10.1002/smll.202409743

**Published:** 2025-01-19

**Authors:** Bixian Ying, Zhenjie Teng, Anatoliy Senyshyn, Maxim Avdeev, Adrian Jonas, Jiali Peng, Søren Bredmose Simonsen, Sylvio Indris, Oleksandr Dolotko, Richard Schmuch, Peng Yan, Michael Merz, Peter Nagel, Stefan Schuppler, Helmut Ehrenberg, Martin Winter, Karin Kleiner

**Affiliations:** ^1^ MEET Battery Research Center University of Muenster 48149 Muenster Germany; ^2^ Heinz Maier‐Leibnitz Centre Technical University of Munich 85747 Garching Germany; ^3^ Australian Centre for Neutron Scattering Australian Nuclear Science and Technology Organisation New Illawarra Rd, Lucas Heights Sydney NSW 2234 Australia; ^4^ School of Chemistry The University of Sydney Sydney NSW 2006 Australia; ^5^ Physikalisch‐Technische Bundesanstalt (PTB) Abbestr. 2–12 10587 Berlin Germany; ^6^ Institute for Applied Materials Karlsruhe Institute of Technology 76344 Eggenstein‐Leopoldshafen Germany; ^7^ Department of Energy Conversion and Storage DTU Energy Technical University of Denmark Kongens Lyngby 2800 Denmark; ^8^ Fraunhofer Research Institution for Battery Cell Production FFB University Muenster 48165 Münster Germany; ^9^ Helmholtz‐Institute Muenster IEK‐12 Research Institute in Juelich 48149 Muenster Germany; ^10^ Institute for Quantum Materials and Technologies Karlsruhe Institute of Technology 76021 Karlsruhe Germany; ^11^ Karlsruhe Nano Micro Facility Karlsruhe Institute of Technology 76344 Eggenstein‐Leopoldshafen Germany

**Keywords:** B‐doping, cathode material, lithium‐ion battery

## Abstract

Rechargeable lithium‐ion batteries (LIBs) are critical for enabling sustainable energy storage. The capacity of cathode materials is a major limiting factor in the LIB performance, and doping has emerged as an effective strategy for enhancing the electrochemical properties of nickel‐rich layered oxides such as NCM811. In this study, boron is homogeneously incorporated into the tetrahedral site of NCM811 through co‐precipitation, leading to an inductive effect on transition metal (*TM*)‐O‐B bonds that delayed structural collapse and reduced oxygen release. Consequently, these changes culminate in an enhancement of cycling performance, translating to an initial specific capacity of 210 mAh g^−1^ and a 95.3% capacity retention after 100 cycles. These interesting findings deepen the understanding of boron doping and shed light on the design of better lithium cathode materials on an applicable scale.

## Graphical abstract

Boron can be homogeneously incorporated onto the tetrahedral site of NCM811 through co‐precipitation, causing an inductive effect on transition metal‐O‐B bonds. This reduces oxygen release, and enhances cycling performance, achieving 210 mAh g−¹ initial capacity and 95.3% retention after 100 cycles. These findings advance boron doping insights and guide cathode design for lithium‐ion batteries.

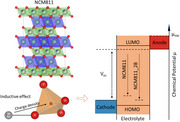

## 1 Introduction

Rechargeable lithium‐ion batteries (LIBs) are a key enabling technology in the pursuit of sustainable electrochemical energy storage.^[^ ^1^ ^]^ The durability of the cathode materials is the key factor limiting the performance of the LIBs.^[^ ^2^ ^]^ Hence, layered transition metal oxides (Li*TM*O_2_), such as Ni‐rich NCMs (LiNi*_x_* Co*_y_* Mn*_1‐x‐y_* O_2_, 0 ≤ *x*, *y* ≤ 1.0; *x* ≥ 0.8) are at the forefront of research due to their high specific capacities (up to 220 mAh g^−1^) at attractive and manageable cell voltages.^[^ ^3^ ^]^ However, rapid capacity and voltage fade, poor thermal stability, and irreversible reactions at the electrode‐electrolyte interfaces still affect their application in today's and next‐generation Li‐ion batteries.^[^ ^4^ ^]^ Effective optimization strategies encompass various techniques, such as elemental cation doping (e.g., Mg,^[^ ^5^ ^]^ Al,^[^ ^6^ ^]^ Ti,^[^ ^6^, ^7^ ^]^ Zr^[^ ^8^ ^]^), and surface coating (B,^[^ ^9^ ^]^ P,^[^ ^10^ ^]^ Cl^[^ ^11^ ^]^). Among others, boron coating has been commonly employed to considerably enhance the performance of NCMs.^[^ ^9b,c^ ^]^ Despite the widespread use of boron‐coated Ni‐rich layered oxides in industrial applications, the underlying mechanism responsible for the enhancing effect of boron coating on lithium‐layered oxides has not been thoroughly studied and understood. In lithium‐layered oxides, Li et al.^[^ ^9c^ ^]^ demonstrated that boron incorporation diminishes the transition metal (*TM*)‐O covalency in Li‐rich materials, thereby inducing alterations in the electronic structure. Moreover, Park et al.^[^ ^9a^ ^]^ and Ryu et al.^[^ ^12^ ^]^ showed that boron incorporation reduces the surface energy of the (003) plane, leading to preferential growth in this plane. Amalraj et al.^[^ ^13^ ^]^ employed Density Functional Theory (DFT) calculation to affirm that the tetrahedral position in the Li layer, i.e., the interstitial position in the Li diffusion pathway is the favored site for boron incorporation.

Additionally, several studies have employed X‐ray diffraction to ascertain the boron occupancy within the rhombohedral structure.^[^ ^9^, ^14^ ^]^ However, due to the limited sensitivity of X‐ray diffraction to light elements like boron,^[^ ^15^ ^]^ these results are not convincing enough. To address this limitation, we synthesized homogeneous bulk doped (B)_Tetra_‐(LiNi_0.8_Mn_0.1_Co_0.1_O_2_)_Octa_ materials via a co‐precipitation method, where “Tetra” signifies tetrahedral site, and “Octa” indicates octahedral site. These materials contained varying amounts of boron at a tetrahedral site, designated as NCM811_xB (x = 0, 0.5, 1, 2, 5 at. %). Neutron diffraction (ND) was adopted to provide robust evidence to support the occupancy of boron at a tetrahedral position. Furthermore, we utilized Near Edge X‐ray Absorption Finestructure Analysis Spectroscopy (NEXAFS) to verify the decreased *TM*‐O covalency resulting from the inductive effect of the B‐O bond. Notably, the synthesis employed in this work can be easily scaled up for large‐scale production, opening promising avenues for the development of high‐performance lithium‐ion batteries on an industrial scale. Boron‐doped NCM811 materials show enhanced capacities, improved rate performances, and increased cycling stabilities compared to conventional NCM811 counterparts due to the inductive effect on *TM*‐O‐B bonds.

## 2 Results and Discussion

### 2.1 Structural Characterization and Analysis

NCM811_xB (x = 0, 0.5, 1, 2, 5 at. %) materials with varying amounts of boron at the tetrahedral site were successfully synthesized using the conventional co‐precipitation method which offers the potential of scale up for industrial applications. The boron contents in the synthesized materials were determined by inductively coupled plasma optical emission spectroscopy (ICP‐OES). In more detail, the co‐precipitation process involved the use of a 5 at. % boric acid solution (0.075 m, calculated based on a 1.5 m transition metal solution) to synthesize NCM811_2B, while for NCM811_5B, a saturated boric acid solution (≈0.8 m at room temperature) was employed. In theory, this method should yield a precursor with ≈50 at. % B‐content. However, in practice, we encountered a limitation, resulting in a precursor with only 5 at. % B‐content. Notably, we observed that the boron content in NCM811 cannot be increased beyond 5 at. %. Additionally, boron incorporation does not follow a linear relationship with the concentration of boric acid, indicating that the doping sites for boron incorporation within the NCM rhombohedral structure are limited.

**Figure 1:**
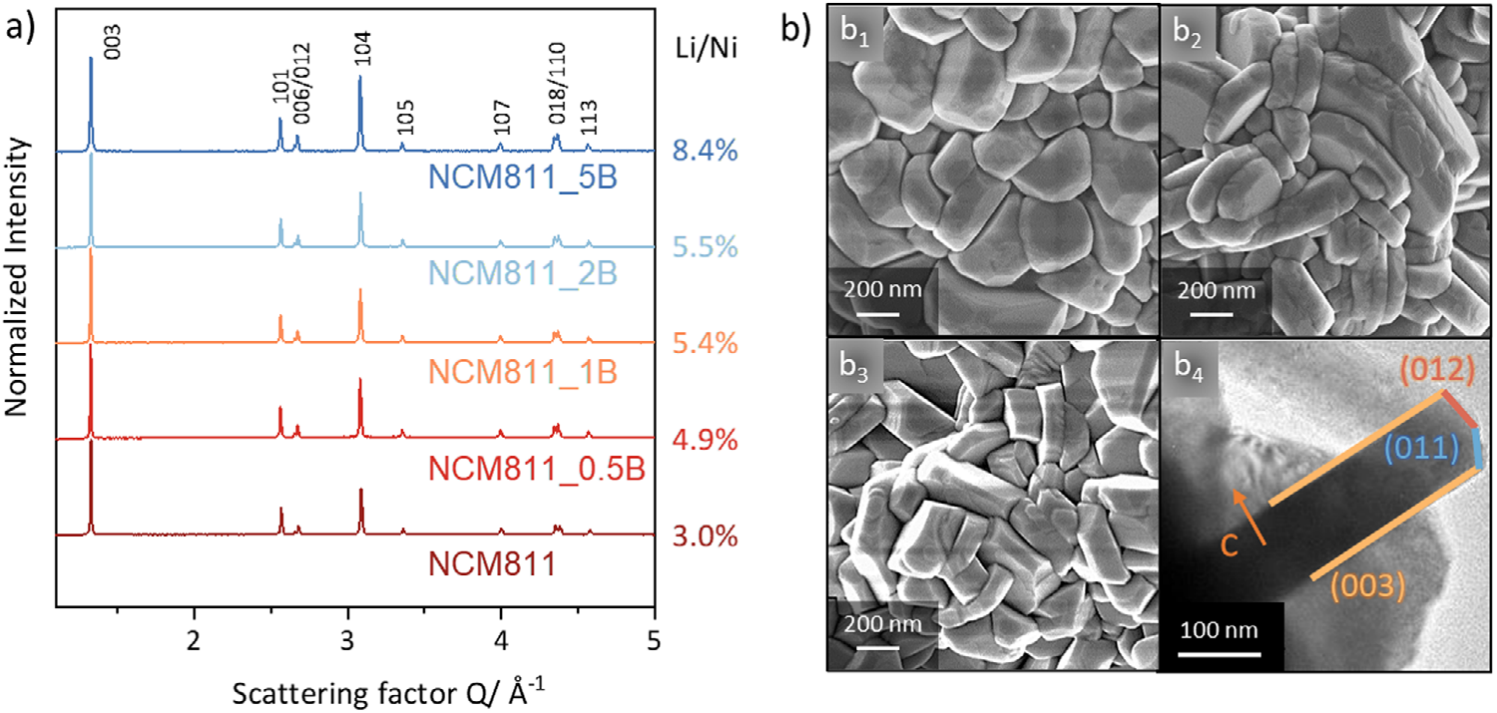
a) XRD Patterns of NCM811_xB, where x = 0, 0.5, 1, 2, 5 at. %, SEM pictures of b_1_) NCM811, b_2_) NCM811_2B and, b_3_) NCM811_5B, b_4_) TEM image of NCM811_5B.

As depicted in **Figure** **1**a, all synthesized materials, including those with different boron concentrations, were found to be phase‐pure with a well‐defined indexed phase of a NaFeO_2_‐type rhombohedral structure, which belongs to the $R\bar{3}m$ space group. The Particle Size Analysis (PSA) results presented in Figure S1 (Supporting Information) reveal a similar mean secondary particle size distribution across all these materials with a close D50 value of 11–12 µm. As more boron is incorporated into the structure, there is an observed trend toward increased lattice parameters (see Table S1, Supporting Information) and a higher Li/Ni disorder (see Figure 1). Li/Ni disorder typically arises from Li/Ni site‐exchange due to their close ionic radii (Li^+^: 0.076 nm, Ni^2+^: 0.069 nm), leading to several issues, such as reduced capacity and structure instability.^[^ ^16^ ^]^ These observations suggest the incorporation of boron into the rhombohedral structure. Additionally, with an increasing boron content, the primary particle size decreases, especially in the c‐direction, as shown in Figure 1b and Figure S2 (Supporting Information). This phenomenon aligns with findings reported by Park et al.,^[^ ^9a^ ^]^ where they suggest that the incorporation of boron results in a decrease of the specific surface energy in the (003) planes, ultimately leading to smaller primary particle sizes.^[^ ^9a^ ^]^ To further validate the successful bulk boron doping, SEM‐EDX analysis performed on a cross‐section of the NCM811_5B sample (Figure S3a, Supporting Information), an XPS depth profile of the B K edge (Figure S3b, Supporting Information) shows that the B 1s signal remains almost constant across the particle although etching might induce B deposits were not expected, which confirmed the homogeneous distribution of boron from the surface to the bulk. Besides, NEXAFS on the B K edge in Figure S4 (Supporting Information) exhibits that the environment around B of NCM811_5B can be comparable to the B environment in B_2_O_3_, H_3_BO_3_, and L_2_B_4_O_7_, with only minor deviations. This further confirms that boron is not only present on the surface but also incorporated within the bulk material.

**Figure 2:**
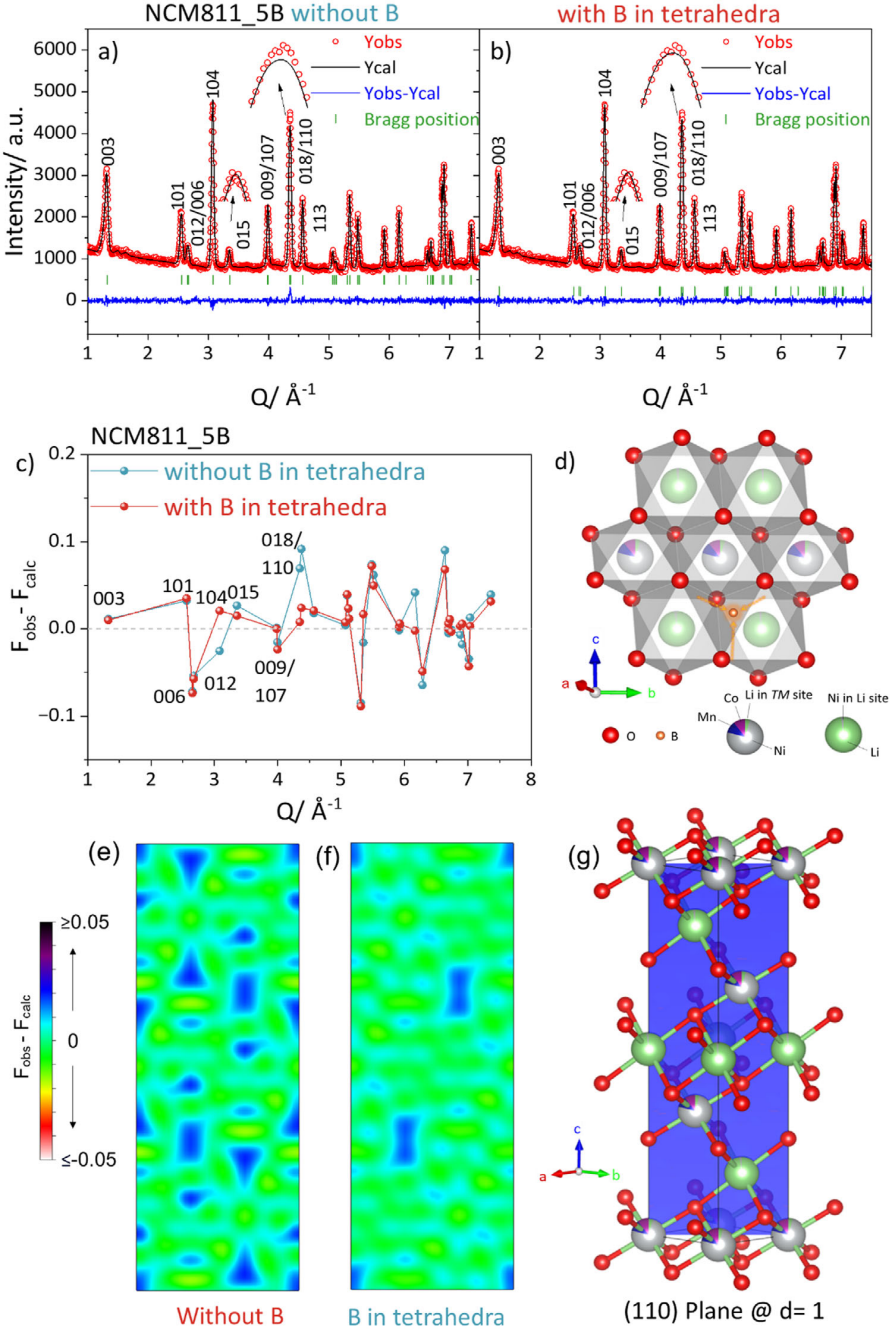
Rietveld refinement against the NCM811_5B neutron diffraction data. a) without B, b) with B at the tetrahedral site, c) F_obs_‐F_calc_ value obtained from Rietveld refinement analysis of NCM811_5B neutron diffraction. d) Schematic illustration shows Li and *TM* octahedral and tetrahedral sites. Difference Fourier maps of (101) plane in NCM811_5B structure, e) without B in the structure, f) with B in tetrahedral site, g) Schematic illustration of (101) plane at d = 1 in NCM811 unit cell, created by VESTA software.^[^ ^21^ ^]^

To investigate the incorporation site of boron, neutron diffraction was conducted on NCM811_5B and NCM811. For the synthesis of NCM811_5B, we utilized the isotope ^11^B to reduce very high neutron absorption of the natural abundance B to enhance the boron signal for more precise detection and analysis. According to the references,^[^ ^17^ ^]^ B^3+^ is incorporated into the tetrahedral interstitial sites of sodium layered oxide due to its relatively small ionic radius of 0.27 Å.^[^ ^18^ ^]^ On the other hand, in the case of lithium layered oxide materials, e.g. NCMs, Li^+^ has an ionic radius of 0.76 Å, while O^2−^ has an ionic radius of 1.26 Å, and transition metal ions like Ni^3+^ have ionic radii ≈0.7 Å.^[^ ^19^ ^]^ This significant difference in ionic radius leads boron to preferentially occupy tetrahedral sites. Moreover, boron has a higher binding energy with oxygen compared to *TM*s in NCM811 (≈809 kJ mol^−1^ for B–O, ≤381 kJ mol^−1^ for Ni–O, ≤403 kJ mol^−1^ for Mn–O, ≤397.4 kJ mol^−1^ for Co‐O).^[^ ^20^ ^]^ This increased covalency in the B‐O bond has the potential to attenuate the covalency of the *TM*‐O bond through an inductive effect, making B^3+^ less likely to substitute Li^+^, *TM* ^n+^, or O^2−^. However, to explore all possibilities, refinements were conducted to examine the potential presence of boron in these sites. Additionally, the tetrahedral interstitial sites were also considered potential incorporation sites for boron. According to the refinement results presented in **Table** **1**, the substitution of Li^+^, *TM* ^n+^, and O^2−^ for B^3+^ can slightly improve the refinement compared to the reference model without boron. However, boron in the tetrahedral sites leads to a significant improvement in the refinement. **Figure** **2**a–c illustrates that with the presence of B in tetrahedral sites, most planes exhibit lower deviations compared to the refinement without B; particularly the (018) and (110) planes are described much better. Schematic depictions of (101) planes with and without B are shown in Figure 2e,f, respectively. Based on the refinement results and the observed improvement in the planes, it can be concluded that boron does not substitute any specific atoms in the NCM811 structure. Instead, it incorporates into the tetrahedral interstitial sites in the Li layers.

**Table 1:** Neutron diffraction refinement results. R_Bragg _: Only points with reflection intensity are considered. R_wp_: weighted R‐factor. χ^2^: goodness of refinement.

NCM811_5B	R_Bragg_	R_wp_	c^2^
Without B	1.916	11.2	1.34
B in Li site	1.745	11.1	1.32
B in *TM* site	2.014	11.3	1.36
B in O site	1.916	11.2	1.35
B in tetrahedral sites	1.357	10.9	1.28
NCM811	R_Bragg_	R_wp_	c^2^
Without B	2.016	10.1	1.40
B in tetrahedra sites	1.970	10.1	1.4

### 2.2 Electrochemical Performance

**Figure 3:**
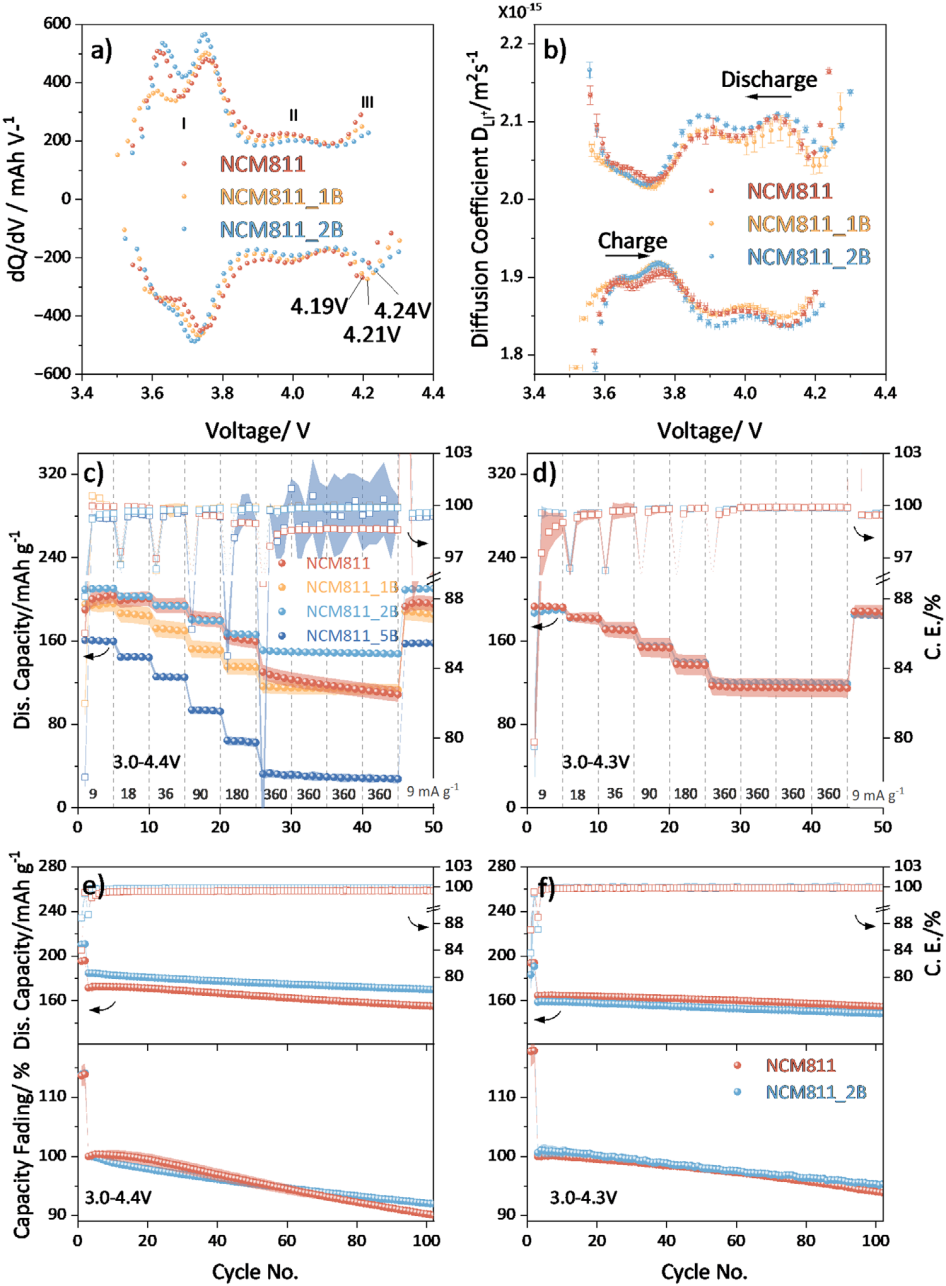
a) The dQ/dV versus voltage curves were derived from GITT data (see Figure S5, Supporting Information), where voltage points are collected after each rest step, b) Lithium diffusion coefficient (D_Li+_) calculated based on GITT data. Rate performance of NCM811_xB versus Li/Li^+^ in the voltage window of c) 3.0–4.4 V, d) 3.0–4.3 V. Cycling stability of NCM811_xB versus Li/Li^+^ in the voltage window of e) 3.0–4.4 V, f) 3.0–4.3 V. Initially, cycling was conducted at a constant current of 9 mA g^−1^ (0.05C‐rate) for the first two cycles, followed by subsequent cycles of 90 mA g^−1^ (0.5C‐rate). b–f) include error bars.

The electrochemical performance of the synthesized NCM811, NCM811_1B, and NCM811_2B is given in **Figure** **3**. To eliminate kinetic influences on the electrochemical data, the dQ/dV versus voltage curves (Figure 3a) were calculated from galvanostatic intermittent titration technique (GITT) measurements by taking the voltage values obtained after each 4‐h relaxation time. A similar change in the peak shape and positions of the dQ/dV versus voltage curves and the diffusion coefficient are found for the reaction peaks I, II, and III. Among these, reaction peak III appears to be more closely linked to anionic redox and oxygen release compared to peaks I and II.^[^ ^22^ ^]^ Thus, this suggests a higher activation barrier for III region due to the presence of boron, which can stabilize the surrounding oxygen atoms by occupying the center of the pseudo‐tetrahedra (Figure 2d). Figure 3b shows the lithium diffusion coefficients calculated from the GITT data. Given that the secondary particle size distributions (Figure S1, Supporting Information) are comparable among the materials, it follows that their diffusion coefficient curves exhibit similar trends.^[^ ^23^ ^]^ Upon charge and discharge, the lithium diffusion coefficient (D_Li+_) follows the dQ/dV versus voltage curves, and the two curves behave opposite to each other. Because the curves behave similarly (but in opposite directions), the analysis primarily concentrates on the discharge. Upon the III reaction region (from 4.4 to 4.2 V), D_Li+_ of NCM811_2B decreases faster than D_Li+_ of NCM811_1B, and NCM811 before reaching a local minimum at ≈4.2 V. This observation leads to the conclusion that at 4.2 V, the near‐collapsed rhombohedral structure of NCM811_2B requires less lithium insertion to stabilize the structure. In other words, its structure is inherently more stable in this reaction peak III voltage range.

NCM811_2B shows the best rate performance and the best initial specific capacity (210 mAh g^−1^) when a voltage window of 3.0–4.4 V (Figure 3c) is applied. In comparison, NCM811 (190 mAh g^−1^), NCM811_1B (196 mAh g^−1^), and NCM811_5B (160.5 mAh g^−1^) show lower initial capacities. It is widely known that the tetrahedral sites, which boron occupies, are part of the lithium diffusion pathway in the structure.^[^ ^24^ ^]^ However, if only a small portion of the tetrahedral sites is occupied, it would not considerably affect the rate capability of the material. Based on the neutron refinement and ICP‐OES, roughly one out of the six available tetrahedral sites in each unit cell is occupied by boron in NCM811_5B. The increased occupancy of tetrahedral sites in NCM811_5B contributes to elevated overpotentials, and higher disorder value (8.4%). These factors are among the reasons that lead to inferior rate performance and reduced capacity compared to other compositions. Within the voltage window of 3.0–4.3 V (Figure 3d), both NCM811_2B and NCM811 have the same initial specific capacity of 175 mAh g^−1^, but the former demonstrates a better rate capability. The cycling stability data in Figure 3e,f shows, that NCM811_2B outperforms NCM811 with a higher cycling stability after 100 cycles (Discharge: 92% vs 89.5%, 95.3% vs 93.1%) in the voltage window of 3.0–4.4 V/4.3 V, respectively.

Initially, NCM811, NCM811_1B, NCM811_2B, and NCM811_5B were cycled within the broader voltage range of 3.0–4.4 V (Figure 3c). NCM811_2B exhibited the highest initial discharge capacity (210 mAh g^−1^) and the best rate performance. However, this upper cut‐off voltage exceeds the typical operating range for NCM811 (≈4.2–4.3 V). Therefore, further tests within the standard voltage range of 3.0–4.3 V (Figure 3d) were conducted only for NCM811_2B and NCM811. These results suggest that the additional capacity of NCM811_2B observed in the 3.0–4.4 V range originates from reaction peak III. This additional capacity can be stably utilized by incorporating boron into the structure.

### 2.3 Inductive Effect

**Figure 4:**
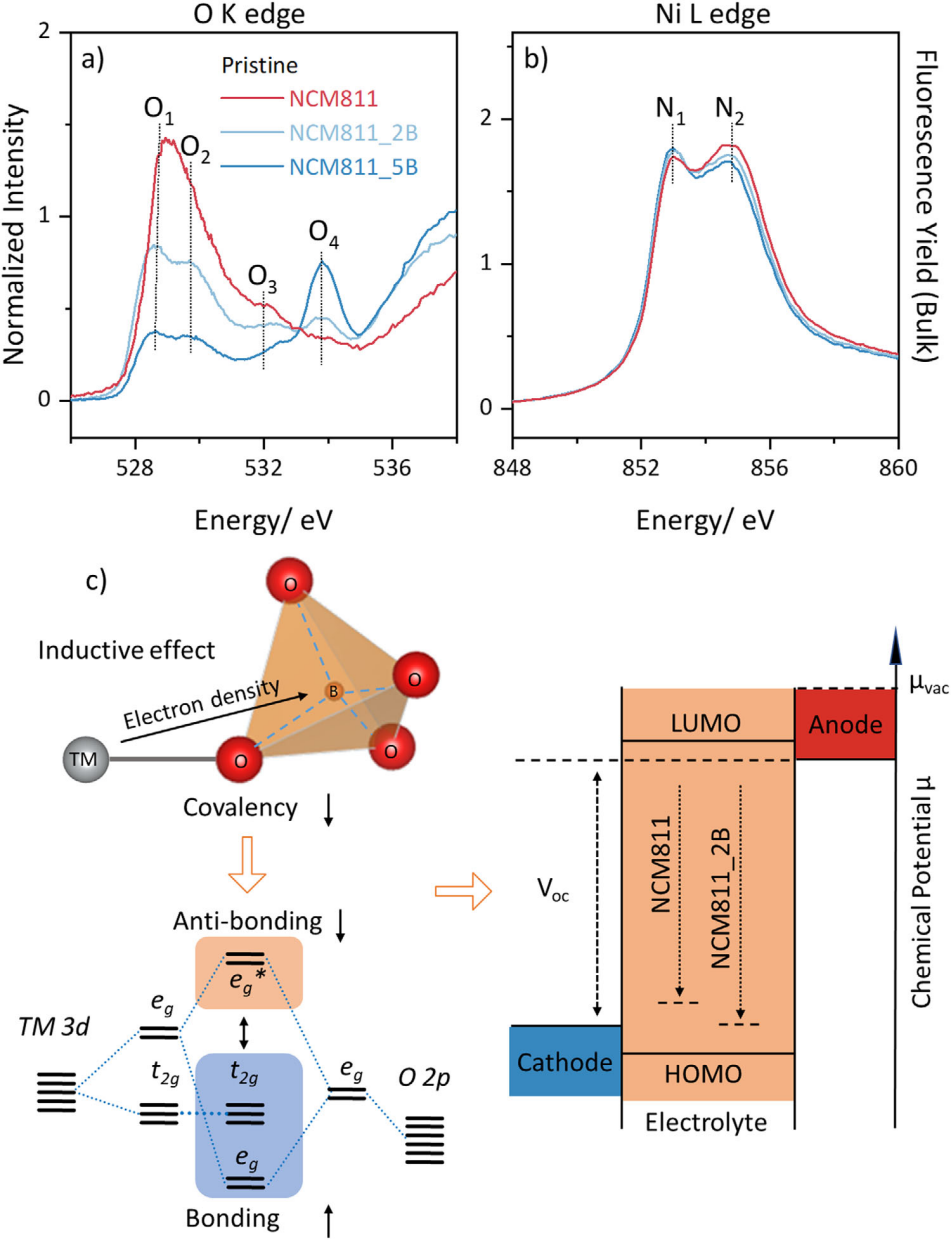
NEXAFS data of the pristine NCM811_xB (x = 0, 2, 5 at. %), a) O K edge and b) Ni L edge c) Bonding energy of *TM*‐O bond is lowered by inductive effect, leading to decreased covalency and a lower chemical potential μ. The electrochemical voltage (V_oc_) is determined by the difference in chemical potential between the anode and the lowest unoccupied 3d metal orbital.

To investigate the mechanism behind the stabilization of the rhombohedral structure through the incorporation of boron in tetrahedral sites (Schematic illustration in Figure 2b), NEXAFS measurements were performed for NCM811, NCM811_2B, and NCM811_5B. NEXAFS is a powerful technique for examining the electronic structure and bonding characteristics of materials. Employing NEXAFS at the *TM* L_2,3_ and O K edges provides valuable information about the oxidation states of 3d metals and O 2p states as well as about the electronic nature of the *TM*–O bonds.^[^ ^25^ ^]^ The NEXAFS measurements were performed in partial fluorescence yield (PFY) mode with a sampling depth of ≈100 nm on the as‐synthesized materials. The normalized data (**Figure** **4**a,b) reveals that B incorporation causes significant variations in O K and Ni L edges. First, as depicted in Figure 4a, an increase in the B content results in a reduced hybridization of Ni and O orbital. This is evident from the changes observed in the O_1_ (≈529 eV) and O_2_ (≈530 V) peaks, which corresponds to a hybridization of Ni t_2_ _g_ or e_g_ orbitals and O 2p orbitals,^[^ ^25a^ ^]^ suggesting a weakening of the Ni and O bonds as well as less overlap and less shared electron density between the atoms. Second, the emergence of a peak at ≈534 eV (O_4_), Figure 4a, which is typically assigned to Hubbard states,^[^ ^25a^ ^]^ signifies a progressive augmentation in electron excitations into the Hubbard band (Discussion about the Hubbard band can be found in Supporting Information O K edge peaks assignment). This strengthening of the Hubbard band indicates localized electronic states near Ni atoms, e.g. characteristic features of ionic Ni^2+^.^[^ ^25a^ ^]^ The 3^rd^ implication from the NEXAFS data is a reduction in the Ni^3+^ content combined with an increase in Ni^2+^ content in Figure 4b. In the Ni L edge, to simplify the interpretation, the N_1_ (≈854 eV) peak corresponds to Ni^2+^, while the N_2_ (≈856 eV) peak is associated with Ni^3+^. According to literature,^[^ ^25^ ^]^ Ni^2+^ is typically associated with a more ionic character, indicating a greater degree of on‐site electron localization from the surrounding oxygen atoms, while Ni^3+^ is considered as more covalent, suggesting a higher degree of electron sharing or delocalization concerning the surrounding oxygen atoms. A decrease in the Ni^3+^ content combined with an increase in the Ni^2+^ content also supports the presence of localized electronic states near Ni atoms. As shown in Figure 4c, the introduction of boron into tetrahedral sites induces an inductive effect on *TM*‐O‐B bonds, as boron shows more electronegativity than *TMs*.^[^ ^26^ ^]^ This leads to an enhanced ionic character of the *TM*‐O bonds and reduces the separation between the bonding and antibonding *TM*‐O orbitals, shifting the reaction peak III to higher voltages, as shown in Figure 3a.

**Figure 5:**
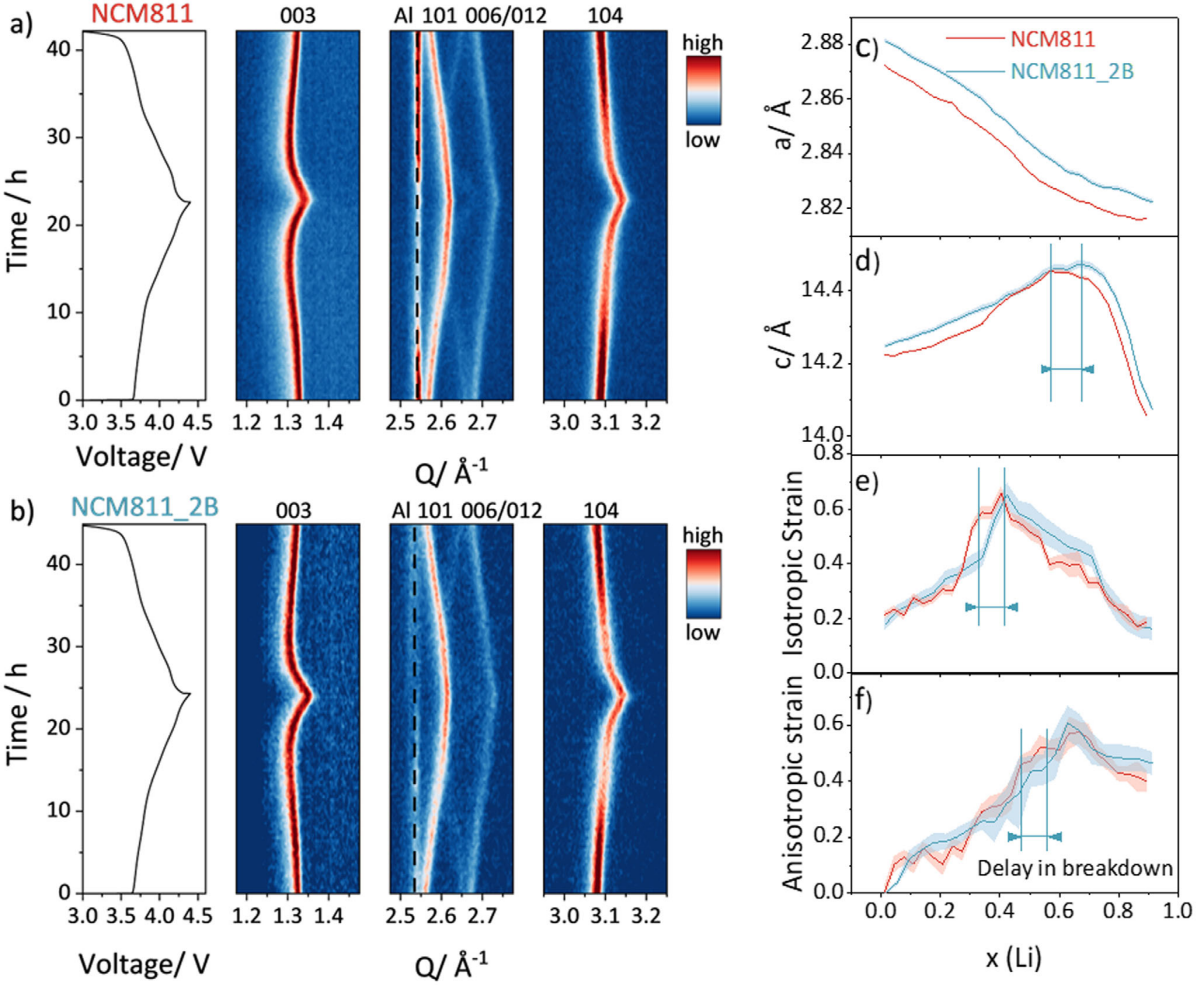
a,b) The contour plot displays the *in operando* XRD data for NCM811 and NCM811_2B at C/20 with a voltage window of 3.0–4 .4V. c–f) The variations in lattice parameters a (= b) and c, isotropic and anisotropic strain values during the initial cycle were determined using Rietveld refinement. The presented values are accompanied by estimated standard deviation. For details about the refinement of isotropic and anisotropic strains, see Supporting Information.

**Figure** **5** shows the *in operando* powder diffraction results of NCM811 and NCM811_2B, along with the Rietveld refinement performed for the discharge to investigate the structural changes. Notably, NCM811_2B exhibits larger initial *a*, *b*, and *c* lattice parameters compared to NCM811. Upon cycling, the *a*, *b* lattice parameter tends to decrease, while the *c* lattice parameter initially increases until it reaches its maximum before starting to decrease.^[^ ^27^ ^]^ The unit cell volume decreases upon cycling. Furthermore, both isotropic anisotropic strains increase, run through a maximum, and later decrease upon further Li extraction. It is evident from the diagrams that the crystallographic changes of NCM811_2B show a delayed response compared to NCM811, indicating the incorporation of boron into the structure contributes to a postponement of the structural collapse, thereby fulfilling a stabilizing role for the overall structure.

**Figure 6:**
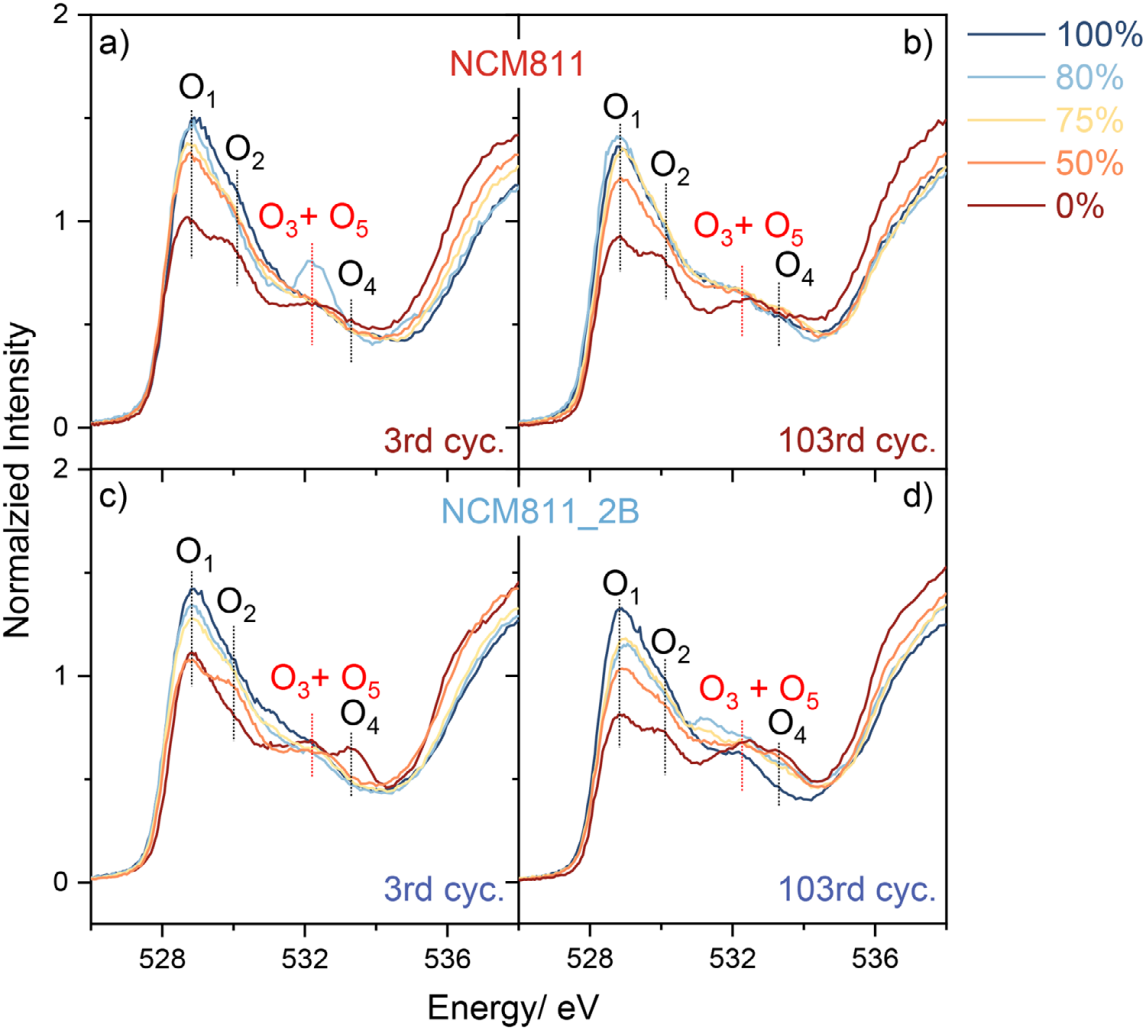
NEXAFS data of cycled samples on O K edge with 0%, 50%, 75%, 80%, and 100% states of charges (SOCs) in partial fluorescence yield. Cycled NCM811 a) at third cycles (cyc.), b) at 103rd cyc. Cycled NCM811_2B c) at 3rd cyc., d) at 103rd cyc.

To investigate the degradation behavior of B‐doped samples, NEXAFS measurements were carried out for NCM811_2B and NCM811 at different SOCs to gain insights into the electronic structural changes upon cycling. The measurements were performed at the 3rd cyc. with C/20, i.e., after 2 formation cycles of C/20; and at the 103rd cyc., i.e., after 2 formation cyc. and 100 cyc. of C/2. Specific states of charges (SOCs) were selected upon discharge, including 0%, 50%, 75%, 80%, and 100% SOC. Particularly, 80% SOC was chosen because it corresponds to the maximum of the reaction peak III dQ/dV versus voltage plot in Figure 3a. Normalized NEXAFS data on the O K edge and the Ni L edge (**Figure** **6**; Figure S6, Supporting Information) showcases the alterations in the electronic structure and characteristics of the material.

Upon the 3rd cycle, both NCM811 and NCM811_2B exhibit an increasing degree of hybridization between Ni and O orbitals with an increasing SOC, as shown in Figure 6 (increasing peaks O_1_ and O_2_). However, at 80% SOC NCM811 demonstrates an increase in the intensity of the O_5_ peak while NCM811_2B does not show this feature. The position of the observed overlapping peak, O_5_, closely resembles that of O_3_ (≈532 eV), and it is associated with the formation of O–O dimers, which corresponds to the release of oxygen^[^ ^22^, ^28^ ^]^ (Discussion about the O_5_ peak assignment can be found in Supporting Information on O K edge peaks assignment). The absence of the O_5_ peak after 100 cycles further supports the association of this peak with oxygen release. This finding indicates that NCM811_2B samples exhibit diminished formation of O–O dimers during cycling when compared to NCM811, which results in less oxygen release and contributes to a more stable cycling performance, as discussed in the **Electrochemical Performance** section. This result is further confirmed by differential electrochemical mass spectroscopy measurements of these two samples in the first cycle (Figure S7, Supporting Information), showing less oxygen evolution in NCM811_2B sample. Thus, we can deduce that the delayed structural collapse and restrained oxygen release during cycling are intricately related to the reinforced oxygen framework through boron incorporation.

### 2.4 Summary and Outlook

In summary, NCM811s with homogeneous bulk boron doping have been successfully synthesized via co‐precipitation. The presence of 2 at. % boron occupying some of the lithium pathway's tetrahedral sites does not hinder the smooth diffusion of Li^+^ within the material, as demonstrated by rate tests. The introduction of boron into tetrahedral sites induces an inductive effect on *TM*‐O‐B bonds, resulting in a reduction of *TM*‐O covalency and strengthening the stability of the oxygen framework within the rhombohedral structure. As a consequence of boron incorporation, structural collapse is postponed and the release of oxygen is diminished, leading to an overall improvement in cycling performance. Based on these findings, it is believed that boron tetrahedral site doping can be universally applicable and serve as a valuable reference for future material research endeavors. The co‐precipitation method used in the synthesis can be adapted for large‐scale production, and therefore, opens up possibilities for the development of high‐performance lithium‐ion batteries on an industrial scale.

## 3 Experimental Section

### Materials Synthesis

The precursors of NCM811 were synthesized using a Couette–Taylor Flow Reactor (Laminar Tera‐3100, Korea) to obtain spherical secondary particle shapes.^[^ ^29^ ^]^ A 1.5 m aqueous solution of metal ions consisting of NiSO_4_·6H_2_O (Acros Organics, purity: 99%), CoSO_4_·7H_2_O (Acros Organics, purity: ≥ 99%), and MnSO_4_·H_2_O (Carl Roth, purity: ≥ 99%) in an 8:1:1 ratio, a 12 wt. % ammonia solution (Acros Organics, purity: ≥ 99%) and a 4.875 m sodium hydroxide solution (Fischer Chemical, purity: 99.44%) were separately and simultaneously pumped into the reactor, respectively. The pH of the mixture is controlled at ≈11. The precipitates were collected after 12 h equilibrium at 60 °C with a stirring speed of 500 rpm. Following co‐precipitation, the precipitates were thoroughly washed with distilled water in Büchner funnel with Büchner flask suction until the pH reached 7. The precipitates were then dried overnight at 80 °C. To obtain the final rhombohedral lithium transition metal oxides (LiNi_0.8_Co_0.1_Mn_0.2_O_2_), the precursor mixed with LiOH∙H_2_O (Fischer Chemical, purity: ≥ 99%) in a molar ratio of 1:1.03 was pre‐heated to 500 °C for 4 h with a heating rate of 2 °C min^−1^ and later calcinated at 800 °C for 10 h with O_2_ flow. In the case of B‐doped NCM811s, homogeneous B‐doped particles were achieved by introducing a boric acid solution into the reactor along with the transition metal solution and base solution. This synthesis method was optimized in a previous publication.^[^ ^19^ ^]^

### Sample Characterization—Scanning Electron Microscopy (SEM)

The morphology of the synthesized precursors and NCMs were examined using scanning electron microscopy (SEM, Carl Zeiss AURIGA, Carl Zeiss Microscopy GmbH) at an accelerating voltage of 3.0 kV and a working distance of 3 mm.

### Sample Characterization—Energy Dispersive X‐Ray Spectroscopy (SEM‐EDX) Cross‐Section

The NCM811_5B electrode was embedded in an epoxy matrix. Once hardened, the sample was cut using Struers cutting machine Accutom‐5, and polished with Struers polishing machine Tegramin‐25. Subsequently, the prepared sample was affixed to a sample holder using silver paste and transferred to the same SEM instrument mentioned earlier. For boron mapping measurements, an accelerating voltage of 3.0 kV and a working distance of 5 mm were employed. Energy dispersive X‐ray spectroscopy was conducted using an X‐MAX 80 mm2 detector to analyze the elemental composition of the samples. The acquired spectra were analyzed using the INCA software developed by Oxford Instruments.

### X‐Ray Diffraction (XRD)

The phase purity of the different NCMs was confirmed with powder diffraction measurements using a Bruker D8 Advance X‐ray diffractometer (Cu Kα_1,2_, λ(K_α1_) = 1.54060 Å, λ(K_α2_) = 1.54443 Å). Additionally, the *in operando* cycling processes of NCM811 and BNCM811 were investigated using a Bruker D8 Advance X‐ray diffractometer with Ag K_α1_ radiation (λ = 0.559410 Å). *In operando* coin cell design: A 5 mm hole has been created on both the negative and positive caps, spacer, and separator to allow the passage of X‐rays (the size of the X‐ray beam ≈1 mm). These two holes on the caps were sealed using Kapton foil by glue. The active material was prepared in the form of a slurry and coated onto a carbon cloth. Celgard2500 separators were utilized.

### Particle Size Analyzer (PSA)

A particle size analysis was performed with a CILAS Particle size analyzer. Inductively coupled plasma optical emission spectroscopy (ICP‐OES): The measurements were performed using an ARCOS spectrometer from SPECTRO Analytical Instruments Company (Germany), with axial plasma viewing, and a standard Fassel‐type torch was utilized.

### Transmission Electron Microscopy (TEM)

A specimen for the TEM study was prepared by grinding the material under ethanol and depositing a few drops of the suspension onto a copper TEM grid covered by a holey carbon layer. TEM images were recorded at 300 kV using a FEI Titan 80–300 microscope.

### X‐Ray Photoelectron Spectroscopy (XPS)

XPS measurements were conducted using a monochromatic Al K_α_ source (hν = 1486.6 eV) with an emission current of 10 mA and an accelerating voltage of 12 kV. To prevent positive charging of the sample surface, a charge neutralizer was employed. The electrode core spectra were acquired in the “hybrid” lens mode, with an analysis area of 2248700 µm × 300 µm. For electrode measurements, the “small area spectroscopy” mode and an aperture were utilized, limiting the analysis area to 110 µm × 110 µm, ensuring that the measurements remained within the sputter crater. In the sputter depth profiling experiments, argon was used as the ion source. Sputtering was performed with a beam energy of 500 eV and an extractor current of 15 µA for various durations (0, 60, 120 s, etc.) to etch layers of the surface or surface contamination.

### Differential Electrochemical Mass Spectrometer (DEMS)

DEMS measurements were performed using a HPR‐20 EGA mass spectrometer (Hiden Analytical Company, UK). The materials were dispersed in a slurry and coated onto an iron mesh. After drying, the electrodes were assembled within a commercial ECC‐DEMS cell (ECC‐Cell Company). The cells were tested using Constant Current Constant Voltage (CCCV) at a 0.1 C‐rate within a voltage window of 3.0–4 .3V.

### Neutron Diffraction (ND)

ND data were collected at the neutron source ECHIDNA, ANSTO (λ = 1.6215 Å).^[^ ^30^ ^]^

### Near Edge X‐Ray Absorption Fine Structures Analysis Spectroscopy (NEXAFS)

To ensure uniform performance, 10 coin cells were assembled for both the 3rd and 103rd cycles. Each electrode's cycling performance was evaluated, and only the most representative electrodes were selected for NEXAFS measurements. Following selection, the electrodes were fully charged and then discharged to a specific state of charge (SOC) using a constant current of C/20. The SOC was determined based on the specific capacity of the previous cycle; for instance, the capacity of the 102nd cycle was used to determine the SOC for the 103rd cycle. Subsequently, the cells were disassembled within the glovebox. Following disassembly, the electrodes were vacuum‐dried overnight in a Buechi Oven. They were then mounted onto the sample plate and transferred to the beamline using a transfer case to mitigate air contamination. NEXAFS data were collected at IQMT's soft X‐ray beamline WERA at the Karlsruhe synchrotron light source KARA (Germany) and PTB (Physikalisch–Technische Bundesanstalt) at the PGM beamline in the PTB laboratory at BESSY II in Berlin, Germany. NEXAFS measurements at the Ni L_2,3_, Co L_2,3_, Mn L_2,3_, O K, and B K were carried out in partial fluorescence yield (PFY) detection mode. The photon‐energy resolution in the spectra was set to 0.2–0.4 eV.

### Electrochemical Characterization

Electrode preparations were performed using 80 wt.% of the NCMs, 10 wt. % C65 (Super C65, Imerys Graphite & Carbon), 10 wt. % polyvinylidene fluoride (PVDF, Kynar flex), and N‐methyl pyrrolidone (NMP, Sigma–Aldrich, 99.5%) as processing solvent. The electrode paste was cast onto an Al‐foil (20 µm thickness, Goodfellow) with a doctor blade (gap height 200 µm, ZAF 2010, Zehntner). The wet film was dried at 80 °C overnight in an oven. 12 mm diameter samples were punched out of the dried electrodes and assembled in a dry room into 2032‐coin cells with two layers of Celgard2500 (16 mm Ø), 36 µL LP572 (1 m LiPF_6_ in ethylene carbonate (EC): ethyl methyl carbonate (EMC), 3:7 by weight, with 2 wt. % vinylene carbonate (VC), BASF, battery grade) and metallic lithium as a counter electrode (15 mm Ø, Albemarle, battery grade). The as‐prepared samples' rate performance, cycling stability, and galvanostatic intermittent titration technique (GITT) are measured using a Maccor Series 4000 automated test system at 20 °C, applying a voltage of 3.0 V–4.3 or 4.4 V. Each electrochemical test involved three coin cells. The electrodes have an average mass loading of ≈6 mg cm^−2^. In specific current and capacity calculations, an active material mass value of ≈4.8 mg cm^−2^ was utilized. A nominal capacity of 180 mAh g^−1^ was used to determine the C‐rates. Consequently, constant currents of 9, 18, 36, 90, 180, and 360 mA g^−1^ were applied for C‐rates of 0.05, 0.1, 0.2, 0.5, 1, and 2 C, respectively.

### Statistical Analysis

The X‐ray (XPD) and neutron diffraction (ND) patterns were analyzed using the software package Fullprof (More details refer to the section *XRD pattern analysis*).^[^ ^31^ ^]^ The Ni/Li disorder was calculated from Rietveld refinements (the refined occupancy of Ni in the Li 3b sites/amount of 3a sites ^*^ 100%). The Ni: Co: Mn ratios were restricted at their expected molar ratios (0.8:0.1:0.1 for NCM811). The structural input parameters for NCM811 were obtained from the crystallographic data file.^[^ ^32^ ^]^ The refinement analysis included the estimated standard deviations of the lattice parameters, which were adjusted by multiplying them with the correlated residuals.^[^ ^33^ ^]^ Differential Fourier maps were obtained from the analyzed data via VESTA.^[^ ^21^ ^]^ NEXAFS data energy calibration (using a NiO reference), dark current subtraction, division by I0, background subtraction, data normalization, and absorption correction were performed as described in the literature.^[^ ^34^ ^]^ For electrochemical characterization, each test was conducted using three coin cells, with error bars represented in the corresponding diagrams.

## Conflict of Interest

The authors declare no conflict of interest.

## Supporting information



Supporting Information

## Data Availability

The data that support the findings of this study are available in the supplementary material of this article.
