# Supplementary material for: Insights into Homogeneous Bulk Boron Doping at the Tetrahedral Site of NCM811 Cathode Materials: Structure Stabilization by Inductive Effect on TM‐O‐B Bonds
Source: Small. 2025 Jan 19;21(9):2409743. doi: 10.1002/smll.202409743 (PMC11878253; doi:10.1002/smll.202409743)
Supplement: Supplementary file 1 — Supporting Information [file SMLL-21-2409743-s001.docx]

# **Insights into Homogeneous Bulk Boron Doping at the Tetrahedral Site of NCM811 Cathode Materials: Structure Stabilization by Inductive Effect on *TM*-O-B Bonds**

Bixian Ying^a*^, Zhenjie Teng^a^, Anatoliy Senyshyn^b^, Maxim Avdeev^c,d^, Adrian Jonas^e^, Jiali Peng^f^, Søren Bredmose Simonsen^g^, Sylvio Indris^f^, Oleksandr Dolotko^f^, Richard Schmuch^h^, Peng Yan^i^, Michael Merz^j, k^, Peter Nagel^j, k^, Stefan Schuppler^j, k^, Helmut Ehrenberg^f^, Martin Winter^a, i^, & Karin Kleiner^a*^

Address

*^a^ MEET, Battery Research Cetre, University of Muenster, 48149 Muenster, Germany*

*^b^ Heinz Maier-Leibnitz Centre, Technical University of Munich, 85747 Garching, Germany*

*^c^ Australian Centre for Neutron Scattering, Australian Nuclear Science and Technology Organisation, New Illawarra Rd, Lucas Heights NSW 2234 Sydney, Australia*

*^d^ School of Chemistry, The University of Sydney, NSW 2006 Sydney, Australia*

*^e^ Physikalisch-Technische Bundesanstalt (PTB), Abbestr. 2-12 10587 Berlin, Germany*

*^f^ Institute for Applied Materials, Karlsruhe Institute of Technology, 76344 Eggenstein-Leopoldshafen, Germany*

*^g^* [*Department of Energy Conversion and Storage*](https://orbit.dtu.dk/en/organisations/department-of-energy-conversion-and-storage)*, DTU Energy,*[*Technical University of Denmark*](https://www.google.com/search?rlz=1C1VDKB_zh-cnDE1019DE1019&sxsrf=APwXEdeAkpImi2Fj4L5pq90uiPyRPwrGdg:1685902302127&q=Technical+University+of+Denmark&ludocid=14917149974382364357&gsas=1&lsig=AB86z5V5li95W7KZrPc_Mc7vi1wI&sa=X&ved=2ahUKEwjVmuC-m6r_AhU2QPEDHfM4AT8Q8G0oAHoECCsQAQ)*, 2800 Kongens Lyngby, Denmark*

*^h^ Fraunhofer Research Institution for Battery Cell Production FFB, University Muenster, 48165 Münster, Germany*

*^i^ Helmholtz-Institute Muenster, IEK-12, Research Institute in Juelich, 48149 Muenster, Germany*

*^j^ Institute for Quantum Materials and Technologies, Karlsruhe Institute of Technology, 76021 Karlsruhe, Germany*

*^k^ Karlsruhe Nano Micro Facility, Karlsruhe Institute of Technology, 76344 Eggenstein-Leopoldshafen, Germany*

* Corresponding author. University of Muenster, Germany

E-mail address: [bying@uni-muenster.de](mailto:bying@uni-muenster.de); karin.kleiner@uni-muenster.de

Supporting Information

**Details about Rietveld refinement:**

**Reflection broadening:**

Regarding the refinement process, the broadening of reflections is dissected into two components: instrumental and sample-related. The instrumental aspect is characterized using a Gaussian function (${FWHM}^{2}(Gaussian)=U\cdot{tan}^{2}(\theta)+V\cdot tan(\theta)+W$) wherein U, V, and W parameters have no physical meaning and are derived from standard material. The sample contribution adheres to a Lorentzian shape and is defined in terms of X (strain broadening) and Y (particle size broadening) of the reflections, as expressed by Eq. 1

|  | $FWHM(lorentzian) = X tan(\theta) +\frac{Y}{cos\left( \theta\right)}$ | Eq. 1 |
| --- | --- | --- |

Anisotropic strain broadening arises from fluctuations in parameters, *e.g.* a variation in $d$-spacing^[^[^1^](#_ENREF_1)^]^. Eq.1 is thus modified by an additional term $F_{SZ}$ to account for anisotropic size broadening (Eq.2)

|  | $FWHM(lorentzian) = X tan(\theta) +\frac{Y+ F_{SZ}}{cos\left( \theta\right)}$ | Eq. 2 |
| --- | --- | --- |

Assuming that the variation of the $d$-spacing ($\frac{1}{d^{2}}=M_{hkl}=\alpha_{1}h^{2}+\alpha_{2}k^{2}+\alpha_{3}l^{2}+\alpha_{4}kl+\alpha_{5}hl+\alpha_{6}hk$ with $h, k, l$*,* the Miller indices and $\alpha_{i}$, the metric parameters of the reciprocal lattice) follows a Gaussian distribution the anisotropic contribution is a function of the variance $\sigma^{2}$ using Bragg´s law.

|  | $F_{SZ}=\sqrt{\sigma^{2}} +d^{2}tan(\theta)$ | Eq. 3 |
| --- | --- | --- |

The variation $\sigma^{2}$, in turn, is governed by the variance of ${\frac{1}{d^{2}}=M}_{hkl}$ and elementary statistics gives Eq.4.

|  | $\sigma^{2}(M_{hkl})=\sum_{ij} \left( C_{ij}\frac{\partial M_{hkl}}{\partial\alpha_{i}}\cdot\frac{\partial M_{hkl}}{\partial\alpha_{j}} \right)=\sum_{HKL} \left( S_{HKL}h^{H}k^{K}l^{L} \right)$ | Eq. 4 |
| --- | --- | --- |

with $C_{ij}=\left\langle{(\alpha}_{i}-\left\langle\alpha_{i} \right\rangle) (\alpha_{j}-\left\langle\alpha_{j} \right\rangle) \right\rangle$, the covariance matrix, $\left\langle\alpha_{j} \right\rangle$, the mean value of the metric parameter, $S_{HKL}$ the strain parameters and $H+K+L=4$. Note that $\frac{\partial M_{hkl}}{\partial\alpha_{1}}= h^{2}, \frac{\partial M_{hkl}}{\partial\alpha_{2}}= k^{2},$… , $\frac{\partial M_{hkl}}{\partial\alpha_{6}}= hk$. In a rhombohedral symmetry the metric parameter $\alpha_{1}=\alpha_{2}=\alpha_{6}$ and $\alpha_{4}=\alpha_{5}=0$ and thus only three anisotropic strain parameters (${S_{400}, S}_{202}$ and $S_{004}$) exist. In the present case, only $S_{202}$ differs significantly from zero as determined with Rietveld refinement.

**General X-ray powder diffraction refinement analysis:**

The Thompson-Cox-Hastings pseudo-Voigt convoluted with axial divergence asymmetry function was used as the normalized peak shape function. The instrumental reflection broadening parameters (U, V, W) were determined separately by using a LaB_6_ NIST standard. These parameters were included in the refinement using an instrumental resolution file (.irf) file. Upon the refinement, the parameters are refined according to the following sequence:

1. Background interpolation and subtraction were used for the refinements. Therefore we have chosen 30 points on the background.
2. The zero shift was refined and left to be free.
3. In cases where the XRD pattern includes an aluminium (Al) phase (reflections originating from the current collector's Al phase), the corresponding refelctions were omitted during the refinement.
4. The operando cell position was adjusted so that the cathode material was in the center of diffraction by comparing the powder diffraction pattern to a capillary measurement performed prior to the experiment. The zero shift was refined once for the OCV measurements and since the position of the operando cell did not change upon cycling, the zero shift was kept constant.
5. The scale factor was refined and was left to be free.
6. The lattice parameters *a*, and *c* were refined according to the space group.
7. The overall Debye-Waller factor (Bov) was refined.
8. The isotropic strain value X and crystallite size value Y were refined.
9. The Lorentzian contribution, isotropic, and anisotropic strain values were refined.
10. The disorder was refined by introducing two more atoms (Ni in the Li 3b site, and Li in Ni 3a site). The total occupancy was restricted to the stoichiometry (Ni in Ni 3a site + Li in Ni 3a site = 0.6664, Li in Ni 3a site + Ni in Ni 3a site = 0.8333).

**Neutron powder diffraction refinement analysis:**

The NCM811 and NCM811_5B samples underwent initial synchrotron X-ray powder diffraction measurements, and these data were refined to ensure their integrity. Subsequently, structural model parameters were refined based on neutron diffraction data. The Thompson-Cox-Hastings pseudo-Voigt function, convoluted with an axial divergence asymmetry function, served as the normalized peak shape model. The instrumental reflection broadening parameters (U, V, W) were independently determined using a LaB_6_ NIST standard and were then incorporated into the refinement process through an instrumental resolution file (.irf). The absorption coefficient µ was estimated using an online app (<https://webapps.frm2.tum.de/neutroncalc/>).

During the refinement, the parameters underwent optimization in the following sequence:

1. Background interpolation and subtraction were used for the refinements. Therefore we have chosen 30 points on the background.
2. The zero shift was refined and left to be free.
3. The scale factor was refined and was left to be free.
4. The lattice parameters *a=b*, and *c* were refined according to the space group (see Tab. S2 NCM811 parameters) in hexagonal setting.
5. The isotropic Debye-Waller factor for every site was refined.
6. The isotropic strain value X and crystallite size value Y were refined.
7. The disorder was refined by introducing two more atoms (Ni in the Li 3b site, and Li in the Ni 3a site). The total occupancy was restricted to the stoichiometry (Ni in Ni 3a sites + Li in Ni 3a sites = 0.6664, Li in Ni 3a sites + Ni in Ni 3a sites = 0.8333,).
8. The previously refined parameters were fixed and the boron isotope (11B) was introduced in 4 different ways: a. Boron on the Li 3b site, b. Boron on the Ni 3a site, Boron on the O 6c site, d. Boron on two tetrahedral sites (Note that there are two tetrahedral sites in this unit cell. Each has 6 equivalent positions on one 6c-site per unit cell in a hexagonal setting and follows the symmetry operations of $R\bar{3}mH$ space group (see Tab. S2, NCM811with tetrahedra sites).

**O K edge peaks assignment:**

Kuiper, Yoon, van Elp, Kim, Koyama, and Montoro *et al*. ^[^[^2^](#_ENREF_3)^]^ have shown, that the ad-mixing of Li into NiO leads to peaks at around 529 eV and 531 eV in the O K spectra which increase with an increasing Li content. With Ni L edge NEXAFS and “charge transfer multiplet calculations” it can be confirmed that Ni-O hybridization increases with an increasing Li content ^[^[^3^](#_ENREF_10)^]^. Thus, the peaks below 532 eV are assigned to holes in the O 2p band which are present due to Ni 3d - O 2p hybridization (Ni 3d^n^ O 2p^6^ **⇌** Ni 3d^n+1^ O 2p^5^ , O 2p^5^ = hole L in the O 2p band) ^[^[^2f^](#_ENREF_8)^,^ [^2g^](#_ENREF_9)^]^.

There are two possibilities which are discussed in literature regarding the peaks at energies > 532 eV.

1. O-O dimer formation

In Li-rich layered oxides (Li[Li_x_*TM*_1-x_]O_2_), and more recently in Ni-rich materials, as well, a very sharp peak at ≈ 533 eV is found which is assigned to O-O formation (a Pi-state) upon oxidation of the transition metal host structure: Gas analysis (namely DEMS and OEMS ^[^[^4^](#_ENREF_12)^]^, resonant inelastic x-ray scattering (RIXS) ^[^[^5^](#_ENREF_15)^]^, and x-ray photoelectron spectroscopy (XPS) ^[^[^6^](#_ENREF_20)^]^ have confirmed the formation of molecular oxygen at high SOCs of the cathode materials (= highly oxidized state).

1. Hubbard states

Strong Coulombic interactions of the 3d electrons introduce a band gap between occupied and unoccupied 3d *TM* states: Electron hopping of a d electron from one into another 3d site (Fig. I) requires overcoming these Coulombic interactions and thus an activation energy is necessary for the transition of an electron between occupied and unoccupied states (see Fig. II a and b. The occupied states are called lower Hubbard, the unoccupied states upper Hubbard band. In NiO (Fig. III), for example, there are no Ni-O hybrid peaks present present ^[^[^2g^](#_ENREF_9)^]^. The broad, asymmetric O K peak visible at ≈ 534 eV is thus assigned to excitations into the Hubbard states. Hybridization, in turn, leads to additional states between the Hubbard states, Fig. IIc).


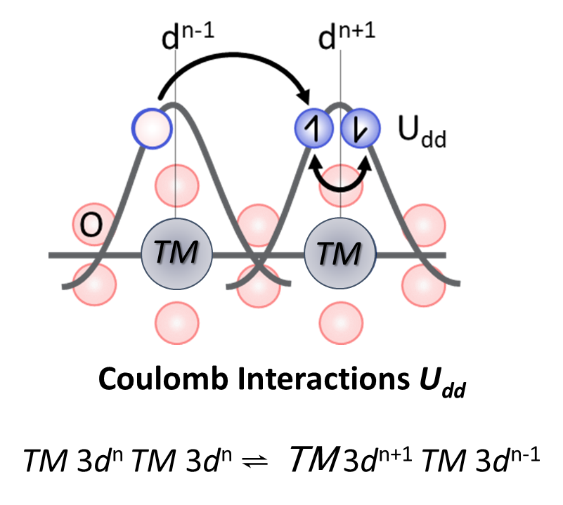


Fig. I Schematic of Coulomb interactions between two *TM*-sites.

The occupied states are called lower Hubbard, the unoccupied states upper Hubbard band. In NiO (Fig. III), for example, there are no Ni-O hybrid peaks present ^[^[^2g^](#_ENREF_9)^]^. The broad, asymmetric O K peak visible at ≈ 534 eV is assigned to excitations into the Hubbard states.


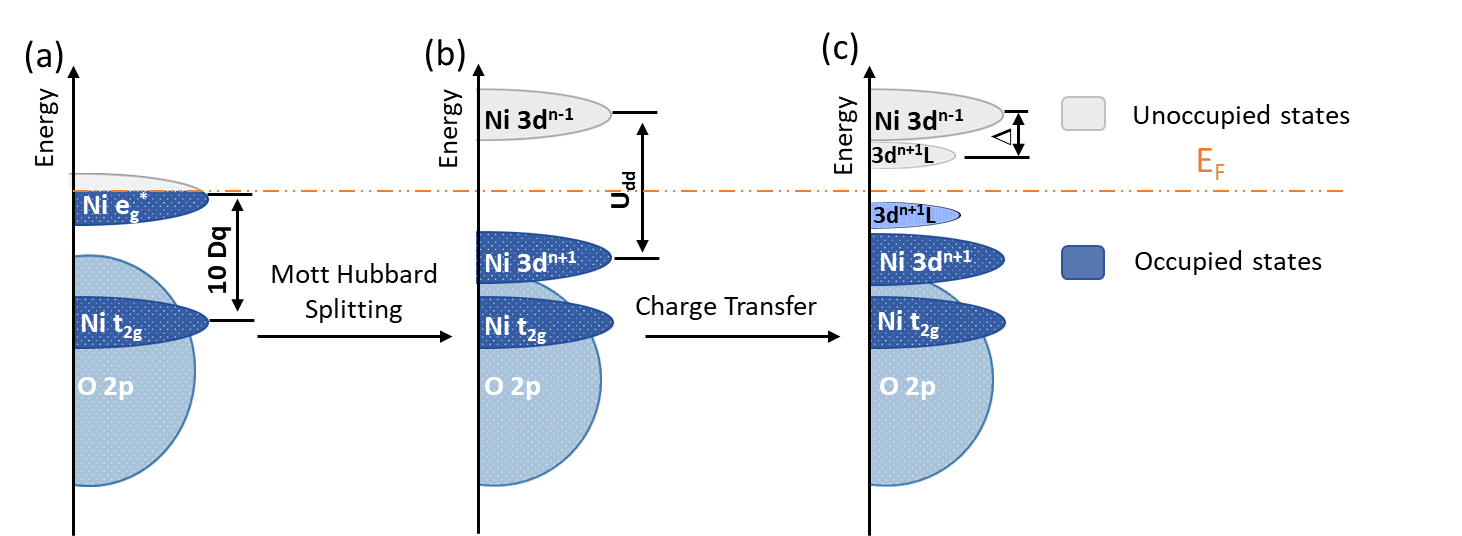


Fig. II Schematic band structure diagrams with Mott-Hubbard splitting and charge transfer. The Mott Hubburd splitting introduces a band gap between occupied and unoccupied states (Fig. IIb). Hybridization, in turn, leads to additional states between the Hubbard states, Fig. IIc.

Fig. III O K edges of NCM811 at different cycle number and of NiO.

In the present manuscript we found a broad O_3_ peak at ≈ 532 eV in the pristine B-NCM811 (Main text, Fig. 4a). The energetic position, the relative broad and asymmetric peak shape as well as the absence of O_2_ formation led us to the conclusion that the origin of the peak is rather of Hubbard type than of O-O dimer formation type. Broader bands observed above 534 eV are associated to transitions into hybridized states of O 2p and higher empty transition metal states such as *TM* 4s - O 2p hybridizations.

Usually, the shoulder peak O_3_ is found in NiO and very ionic Li_x_*TM*O_y_ samples ^[^[^7^](#_ENREF_23)^]^. The presence of O_3_ in ionic NiO as a shoulder peak (Fig. III) shows, that it cannot be attributed to Ni-O hybrid states. In NiO there is also no oxygen evolution or O-O dimer formation. Moreover, systematic studies on ageing of layered oxides show that Li*TM*O_2_ undergoes surface morphology changes of the type Li*TM*O_2_ —> Li_x_*TM*O_2-y_ + y/2 O_2_ + ye^-^. These studies reveal a systematic change in position and intensity of O_3_ with an in/decreasing covalent character of the samples.^[^[^8^](#_ENREF_24)^]^ Similar to the changes observed in the B-doped samples. Although the real origin of the O_3_ peak remains widely unclear, its assignment to a Hubbard band is reasonable considering the absence of *TM*-O hybrid states and oxygen evolution reactions while O_3_ is observed.

Thus. the O_3_ and O_4_ peaks in LiNiO_2_, NCM111 (LiNi_1/3_Co_1/3_Mn_1/3_O_2_), NCM622 (LiNi_0.6_Co_0.2_Mn_0.2_O_2_), and NCM811 (LiNi_0.8_Co_0.1_Mn_0.1_O_2_) are usually attributed to Hubbard bands. With doping, O_3_ and O_4_ significantly change in position and intensity showing that the electronic character of the materials changes from a more “charge transfer type” towards a more “Hubbard” type semiconductor (e.g., O_4_ increases in intensity with an increasing B content, Main text, Fig. 4a). In contrast, charge compensation upon de-lithiation (charge) and lithiation (discharge) of Li*TM*O_2_ changes the Ni-O hybrid states at 529 eV-531 eV with Hubbard bands exhibiting little involvement in electronic changes during cycling (Main text, Fig. 6). O_5_, in turn, just appears at the point upon cycling where oxygen release sets in (80% SOC). It is a relative symmetric peak and lies at energies where usually the O-O peak is observed.^[^[^9^](#_ENREF_26)^]^ Due to its appearance at 80% SOC (reaction peak III) and subsequent disappearance thereafter we assign O_5_ to the oxygen dimer.^[^[^9^](#_ENREF_26)^]^. With boron incorporated into the structure, O_5_ is reduced in intensity and oxygen release is supressed.

Fig. S1 Particle size distribution of NCM811, NCM811_1B, and NCM811_2B.


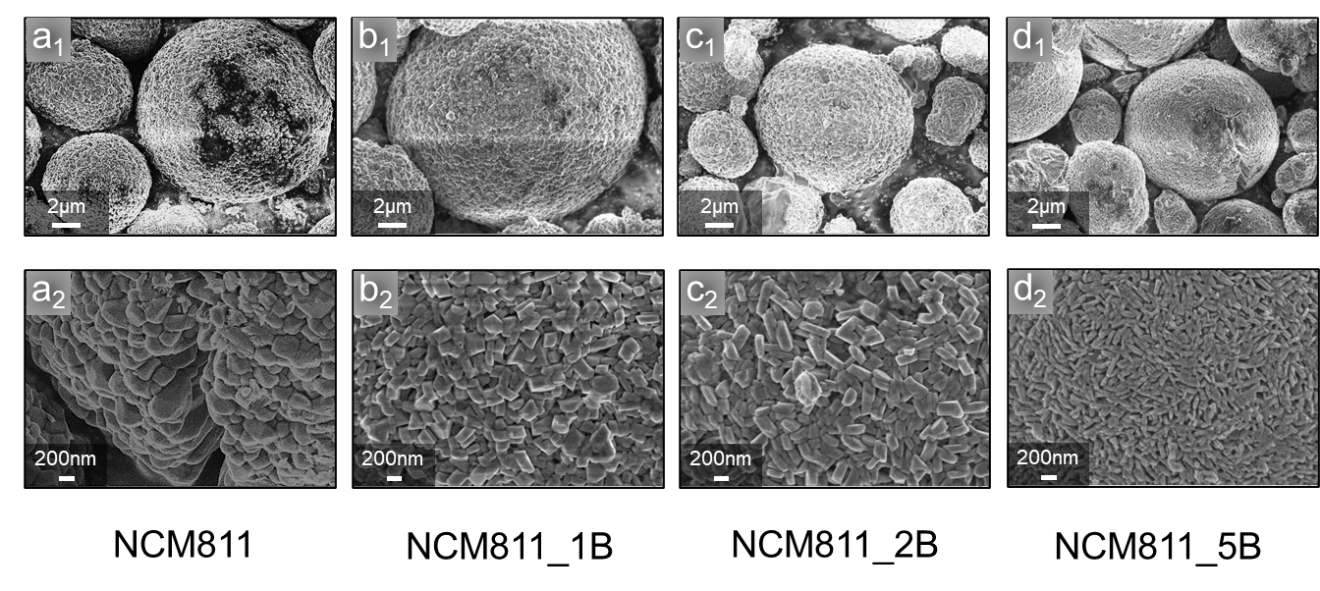


Fig. S2 SEM images of NCM811_xB (x= 0, 0.5, 1, 2, 5%)

Fig. S3 cross-section of NCM811_5B electrode with SEM-EDX measurement. XPS depth profiles of NCM811_5B: b) B 1s, c) O 1s, and Ni 2p edges. Different durations of seconds correspond to varying etching times, enabling the probing of samples at different thicknesses.

Fig.S3 depicts the results of SEM-EDX analysis performed on a cross-section of NCM811_5B, which confirmed the homogeneous distribution of boron within the particles, providing evidence for the successful bulk boron doping. However, it should be noted that due to the similarity in detection energies of boron and carbon, some boron signals may be detected in the matrix, and carbon signals may be detected in the bulk. Nevertheless, the analysis showed that in the bulk region, there is a much higher concentration of boron compared to carbon, while in the matrix region, carbon dominates.

XPS depth profiles of the boron K edge (Fig. S3b-c) show a homogeneous boron distribution from the surface to the bulk. The B 1s signal remains almost constant across the particle although etching might induce B deposits were not expected. In Fig. S3c, at the surface of B-doped material (Etching time = 0 s), a dominant carbonate peak is observed, which is assigned to Li_2_CO_3_ or other carbonate impurities. As etching time increases, this carbonate peak diminishes in prominence, while a peak corresponding to lattice oxygen emerges. The intensity of this lattice oxygen peak stabilizes after an etching time of 300 s, suggesting the removal of nearly all surface species and probing of the bulk material. In Fig. S3d, at the surface (Etching time = 0 s), Ni 2p _3/2_ and Ni 2p _1/2_ peaks appear blurred due to surface impurities. With increasing etching time, both the intensity and resolution of these peaks improve as surface impurities are removed. After an etching time of 300 s, the intensities remain almost constant, further supporting the removal of most surface species and the probing of the bulk material. Therefore, by using these XPS spectra, we demonstrate that we can probe the surface and the bulk material. In addition, B K NEXAFS in FY yield mode was performed to proof the presence Boron in the bulk, Fig. S4.

Fig. S4 NEXAFS spectra of NCM811_5B, B_2_O_3_, H_3_BO_3_, and L_2_B_4_O_7_ (fluorescence yield)

Fig. S5 GITT data of NCM811, NCM811_1B and NCM811_2B. During each charging/discharging step, a constant current of 9 mA g^-1^ (0.05 C-rate) was applied for 0.5 h, followed by a 4-hour rest, continuing until it reached the voltage limit

Fig. S6 NEXAFS data of cycled samples on Ni L edge with 0%, 50%, 75%, 80%, and 100% states of charges (SOCs) in fluorescence yield. Cycled NCM811 a) at 3rd cycle (cyc.), b) at 103rd cyc. Cycled NCM811_2B c) at 3rd cycle (cyc.), d) at 103rd cyc.

Fig. S7 a) voltage *vs.* time curve b) differential capacity (dq/dV) *vs.* time, and c) oxygen evolution (m/z = 32 amu) of NCM811, and NCM811_2B during operando differential electrochemical mass spectroscopy (DEMS) tests. The cell was rested for 2 hours under an Ar flow to prevent oxygen contamination during the connection and allow for baseline substraction.

Tables:

To give the reader a better understanding of how the refinement of the data was performed, the following tables give parameters used and obtained upon data processing. R_Bragg_ values are given for the most important refinements. They reflect the difference between the observed and calculated structure factors normalized to the observed values, multiplied by their multiplicity. Note that Rp values are not actively included to minimize mismatches in the refinement. The table also includes the estimated standard deviations of lattice parameters obtained from the Rietveld refinement analysis. These standard deviations have been multiplied by correlated residuals^[^[^10^](#_ENREF_30)^]^.

Tab.S1 Refinement analysis results of XRD (Cu source) for NCM811_xB (x= 0, 0.5, 1, 2, 5%)

| NCM811_xB | a = b lattice parameter | c lattice parameter | R_Bragg_ |
| --- | --- | --- | --- |
| 0B | 2.8675(1) | 14.1955(11) | 2.01 |
| 0.5B | 2.8700(1) | 14.1956(11) | 2.82 |
| 1B | 2.8721(1) | 14.1963(12) | 1.63 |
| 2B | 2.8734(1) | 14.1967(10) | 1.71 |
| 5B | 2.8746(1) | 14.1985(11) | 1.23 |

Tab.S2 Crystallographic parameters used in this work.

| LiNi_0.8_Co_0.1_Mn_0.1_O_2_ Cell parameters^[^[^11^](#_ENREF_32)^]^ | | | | | |
| --- | --- | --- | --- | --- | --- |
| Space group: *R-3mH*, *a* = *b* = 2.8645 Å, *c* = 14.161 Å, gamma = 120°, V = 100.63 Å^3^, *Z*= 3 | | | | | |
| Atomic positions | | | | | |
| Name | site | x | y | z | Fract. |
| Li1 | 3b | 0.00 | 0.00 | 0.50 | 0.98(1) |
| Ni1 | 3b | 0.00 | 0.00 | 0.50 | 0.02(1) |
| Li2 | 3a | 0.00 | 0.00 | 0.00 | 0.05(2) |
| Ni2 | 3a | 0.00 | 0.00 | 0.00 | 0.75(2) |
| Co1 | 3a | 0.00 | 0.00 | 0.00 | 0.10 |
| Mn1 | 3a | 0.00 | 0.00 | 0.00 | 0.10 |
| O1 | 6c | 0.00 | 0.00 | 0.259(1) | 1.00 |
| LiNi_0.8_Co_0.1_Mn_0.1_O_2_B_0.05_ Cell parameters | | | | | |
| Space group: *R-3mH*, *a* = *b* = 2.8645 Å, *c* = 14.161 Å, gamma = 120°, V = 100.63 Å^3^, *Z*= 3 | | | | | |
| Atomic positions |  |  |  |  |  |
| Name | site | x | y | z | Fract. |
| Li1 | 3b | 0.00 | 0.00 | 0.50 | 0.98(1) |
| Ni1 | 3b | 0.00 | 0.00 | 0.50 | 0.02(1) |
| Li2 | 3a | 0.00 | 0.00 | 0.00 | 0.05(2) |
| Ni2 | 3a | 0.00 | 0.00 | 0.00 | 0.75(2) |
| Co1 | 3a | 0.00 | 0.00 | 0.00 | 0.10 |
| Mn1 | 3a | 0.00 | 0.00 | 0.00 | 0.10 |
| O1 | 6c | 0.00 | 0.00 | 0.259(1) | 1.00 |
| B1 (tetrahedral site) | 6c | 0.00 | 0.00 | 0.140 | 0.021 |
| B2 (tetrahedral site) | 6c | 0.00 | 0.00 | 0.285 | 0.029 |


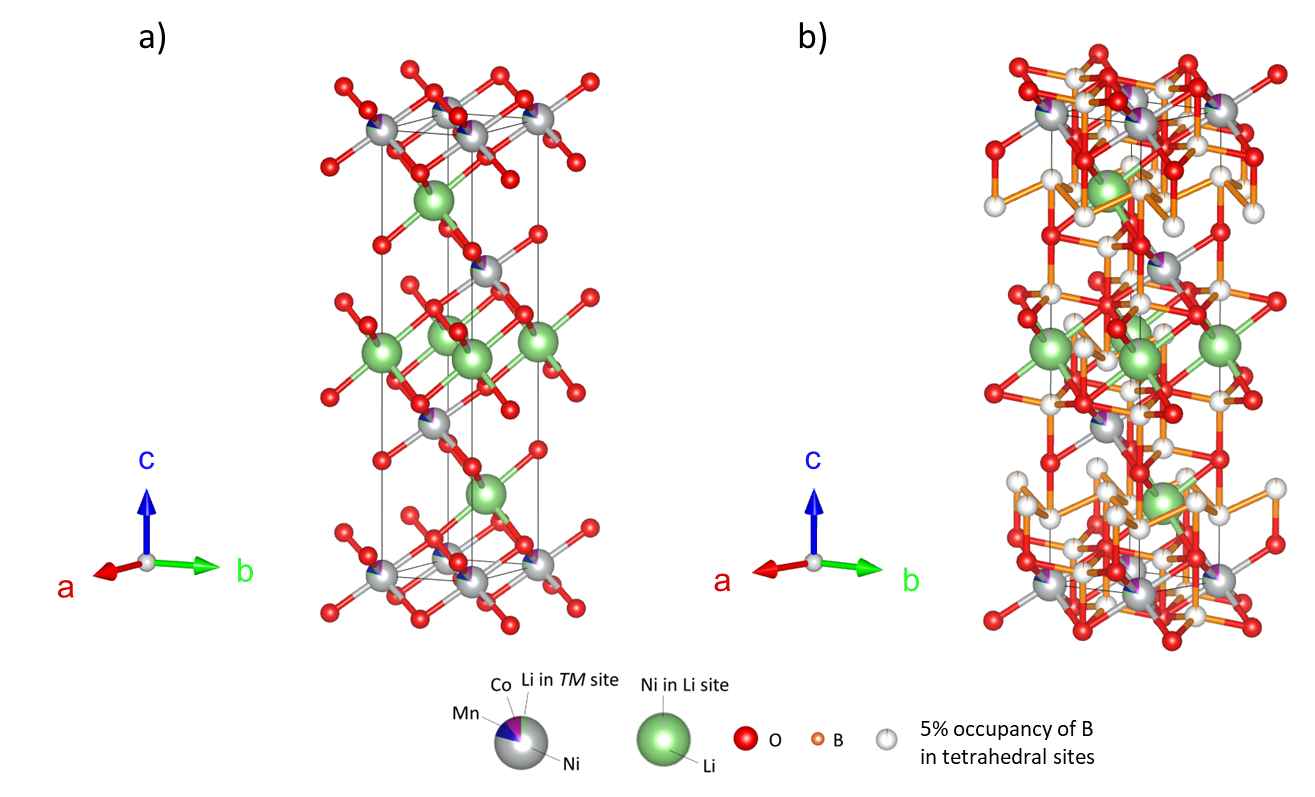


Fig. S8 a) a unit cell of LiNi_0.8_Co_0.1_Mn_0.1_O_2_ and b) a unit cell of LiNi_0.8_Co_0.1_Mn_0.1_O_2_B_0.05_ with boron in two tetrahedral sites.

Reference:

[1] aP. W. Stephens, *Journal of Applied Crystallography* **1999**, *32*, 281-289; bJ. Rodriguez-Carvajal, M. Fernandez-Diaz, J. Martinez, *Journal of Physics: Condensed Matter* **1991**, *3*, 3215.

[2] aW.-S. Yoon, K. Y. Chung, J. McBreen, X.-Q. Yang, *Electrochemistry Communications* **2006**, *8*, 1257-1262; bW.-S. Yoon, K. Y. Chung, J. McBreen, D. A. Fischer, X.-Q. Yang, *Journal of Power Sources* **2007**, *174*, 1015-1020; cM. G. Kim, N. E. Sung, H. J. Shin, N. S. Shin, K. S. Ryu, C. H. Yo, *Electrochimica acta* **2004**, *50*, 501-504; dY. Koyama, T. Mizoguchi, H. Ikeno, I. Tanaka, *The Journal of Physical Chemistry B* **2005**, *109*, 10749-10755; eL. Montoro, J. M. Rosolen, *Electrochimica Acta* **2004**, *49*, 3243-3249; fP. Kuiper, G. Kruizinga, J. Ghijsen, G. A. Sawatzky, H. Verweij, *Physical review letters* **1989**, *62*, 221; gJ. Van Elp, H. Eskes, P. Kuiper, G. Sawatzky, *Physical Review B* **1992**, *45*, 1612.

[3] aL. Montoro, M. Abbate, E. Almeida, J. M. J. C. P. L. Rosolen, **1999**, *309*, 14-18; bK. Kleiner, C. A. Murray, C. Grosu, B. Ying, M. Winter, P. Nagel, S. Schuppler, M. Merz, *Journal of The Electrochemical Society* **2021**, *168*, 120533.

[4] aQ. Li, D. Ning, D. Wong, K. An, Y. Tang, D. Zhou, G. Schuck, Z. Chen, N. Zhang, X. J. N. C. Liu, **2022**, *13*, 1123; bT. Teufl, B. Strehle, P. Müller, H. A. Gasteiger, M. A. J. J. o. T. E. S. Mendez, **2018**, *165*, A2718-A2731; cB. Strehle, K. Kleiner, R. Jung, F. Chesneau, M. Mendez, H. A. Gasteiger, M. J. J. o. T. E. S. Piana, **2017**, *164*, A400.

[5] aE. Zhao, Q. Li, F. Meng, J. Liu, J. Wang, L. He, Z. Jiang, Q. Zhang, X. Yu, L. Gu, *Angewandte Chemie International Edition* **2019**, *58*, 4323-4327; bR. A. House, G. J. Rees, M. A. Pérez-Osorio, J.-J. Marie, E. Boivin, A. W. Robertson, A. Nag, M. Garcia-Fernandez, K.-J. Zhou, P. G. Bruce, *Nature Energy* **2020**, *5*, 777-785; cR. A. House, G. J. Rees, K. McColl, J.-J. Marie, M. Garcia-Fernandez, A. Nag, K.-J. Zhou, S. Cassidy, B. J. Morgan, M. Saiful Islam, *Nature Energy* **2023**, *8*, 351-360; dR. A. House, J.-J. Marie, J. Park, G. J. Rees, S. Agrestini, A. Nag, M. Garcia-Fernandez, K.-J. Zhou, P. G. Bruce, *Nature Communications* **2021**, *12*, 2975; eR. A. House, U. Maitra, M. A. Pérez-Osorio, J. G. Lozano, L. Jin, J. W. Somerville, L. C. Duda, A. Nag, A. Walters, K.-J. Zhou, *Nature* **2020**, *577*, 502-508.

[6] aE. McCalla, A. M. Abakumov, M. Saubanère, D. Foix, E. J. Berg, G. Rousse, M.-L. Doublet, D. Gonbeau, P. Novák, G. Van Tendeloo, *Science* **2015**, *350*, 1516-1521; bG. Assat, J.-M. Tarascon, *Nature Energy* **2018**, *3*, 373-386; cG. Assat, D. Foix, C. Delacourt, A. Iadecola, R. Dedryvère, J.-M. Tarascon, *Nature communications* **2017**, *8*, 2219.

[7] K. Kleiner, J. Melke, M. Merz, P. Jakes, P. Nagel, S. Schuppler, V. Liebau, H. Ehrenberg, *ACS applied materials & interfaces* **2015**, *7*, 19589-19600.

[8] aS. Yin, W. Deng, J. Chen, X. Gao, G. Zou, H. Hou, X. J. N. E. Ji, **2021**, *83*, 105854; bB. H. Goodge, D. Li, K. Lee, M. Osada, B. Y. Wang, G. A. Sawatzky, H. Y. Hwang, L. F. J. P. o. t. N. A. o. S. Kourkoutis, **2021**, *118*, e2007683118.

[9] aE. Hu, Q. Li, X. Wang, F. Meng, J. Liu, J.-N. Zhang, K. Page, W. Xu, L. Gu, R. Xiao, *Joule* **2021**, *5*, 720-736; bA. Menon, B. Johnston, S. Booth, L. Zhang, K. Kress, B. Murdock, G. P. Fajardo, N. Anthonisamy, N. Tapia-Ruiz, S. Agrestini, *PRX Energy* **2023**, *2*, 013005; cG. H. Lee, J. Wu, D. Kim, K. Cho, M. Cho, W. Yang, Y. M. Kang, *Angewandte Chemie* **2020**, *132*, 8759-8766; dN. Li, S. Sallis, J. K. Papp, B. D. McCloskey, W. Yang, W. Tong, *Nano Energy* **2020**, *78*, 105365.

[10] aJ.-F. Bérar, P. Lelann, *Journal of applied crystallography* **1991**, *24*, 1-5; bJ. Berar, *Pow. Diff II”, NIST sp. Pub* **1992**, *846*, 63.

[11] H. Arai, M. Tsuda, Y. Sakurai, *Journal of power sources* **2000**, *90*, 76-81.
